# The City Nature Challenge as an urban BioBlitz: evaluating Citizen Science contributions to biodiversity monitoring in Berlin

**DOI:** 10.1186/s12862-026-02524-w

**Published:** 2026-05-20

**Authors:** Julia Flister, Alexis Tinker-Tsavalas, Silke Voigt-Heucke, Boris Schröder, Frederic Griesbaum

**Affiliations:** 1 Museum für Naturkunde - Leibniz Institute for Evolution and Biodiversity Science, Invalidenstraße 43, 10115 Berlin, Germany; 2 Department of Plant Ecology, Technische Universität Berlin, Institute of Ecology, Rothenburgstr. 12, 12165 Berlin, Germany; 3 Leibniz Institute for Zoo and Wildlife Research, Alfred-Kowalke-Straße 17, 10315 Berlin, Germany

**Keywords:** Citizen science, Biodiversity, BioBlitz, Ecology, Conservation, Participation, Monitoring

## Abstract

**Background:**

The human-induced loss of biodiversity demands innovative, resource efficient monitoring approaches, such as Citizen Science, to complement traditional biodiversity monitoring methods. BioBlitz events are an established Citizen Science format for biodiversity monitoring that generates as many species observations as possible in a short period of time and within a limited area through collaboration between scientists, the public and nature enthusiasts. The most prominent example of a large-scale worldwide urban BioBlitz event is the City Nature Challenge (CNC). This study investigates whether and how BioBlitz events, specifically the CNC Berlin, can complement existing biodiversity monitoring by addressing three key questions: (1) how species composition compares to existing datasets, (2) how data quality varies across taxonomic groups and participants, and (3) how participant engagement shapes data generation. We further assess taxonomic biases in the CNC Berlin 2023 and 2024 datasets to evaluate their contribution to urban biodiversity monitoring. For a better understanding, the CNC Berlin data is compared with a reference dataset from GBIF (Global Biodiversity Information Facility), an international open-access database compiling biodiversity records from multiple observation platforms. The reference dataset is limited to Berlin, covers a comparable time period and includes biodiversity data from a number of Citizen Science observation platforms.

**Results:**

Participation in CNC Berlin has increased substantially, with the number of contributors rising from 184 in 2023 to 361 in 2024, reflecting growing involvement. Analysis of the uploaded observations during CNC 2023 and 2024 in Berlin revealed clear differences in participant activity levels: while highly active individuals were few, they contributed a substantial portion of the total observations. The comparison of the observed species in taxonomic groups from CNC Berlin 2023 and 2024 with the GBIF reference dataset revealed the impact of highly active species experts participating in BioBlitz events on particular species groups like fungi and lichens. This highlights the influence of species experts on BioBlitz datasets. In total birds, reptiles, fish, and mammals were more likely to reach “Research Grade” status on iNaturalist, whereas arachnids, protozoans, insects, and chromista had substantially lower Research-Grade rates. Of the 2,440 species listed in the GBIF reference dataset, 1,027 species (42%) were also recorded during CNC Berlin 2023 and 2024. Observations included threatened amphibians, such as the Spadefoot Toad (*Pelobates fuscus*) and the Great Crested Newt (*Triturus cristatus*), rare birds like the Whinchat (*Saxicola rubetra*), and several invasive species, including crayfish (*Procambarus* spp.) and Giant Hogweed (*Heracleum mantegazzianum*). Notably, the potentially invasive mysid *Hemimysis anomala* was recorded in Berlin for the first time ever in 2024. As observations from CNC 2023 had more time to reach “Research Grade” than those from 2024, differences between years should be interpreted with caution.

**Conclusion:**

BioBlitz events and Citizen Science in general, can provide valuable data that can complement traditional biodiversity monitoring and help to close research gaps, particularly by documenting endangered, invasive and non-native species, and other conservation-relevant taxa. We encourage other researchers to investigate large BioBlitz datasets like CNCs in other parts of the world or even comprehensively on a larger geographic scale.

**Supplementary information:**

The online version contains supplementary material available at 10.1186/s12862-026-02524-w.

## Background

The human-induced loss of biodiversity, along with the climate crisis, represents the most important environmental challenges of our time [1, 2]. The increasing decline of biodiversity poses a serious conservation issue and requires innovative approaches to efficiently document populations of rare taxa and areas of high conservation value in order to protect them before populations are lost and taxa become extinct. However, classic long-term biodiversity monitoring is limited in terms of resources like the involvement of professional species experts and funding [3] and hence spatial and temporal coverage. Therefore, less resource-intensive and efficient methods of biodiversity monitoring are needed.

### Citizen Science & BioBlitz events

One such approach is Citizen Science, a participatory research method that has become a valuable complement to traditional standardised biodiversity monitoring methods related to monitoring of species occurrences and future distribution patterns (e.g. [4–7]). Citizen Science is already recognized as a powerful tool for biodiversity research and monitoring [8–10] and integrating various survey techniques into a comprehensive monitoring scheme has recently been promoted by many authors (e.g. [11–13]). BioBlitz events are a well-established Citizen Science format for biodiversity monitoring, which, despite varying widely in their structure and organization [14], always aim to gather as many species observations as possible within a defined area and time frame [15]. They consistently involve collaboration between scientists, naturalists, and the public [16, 17], often with Natural History Museums as connecting institutions [18], although there are also so-called expert BioBlitzes in which only scientists participate [19]. While Citizen Science projects in general can strengthen ecological awareness, deepen knowledge of environmental issues [20, 21], and even encourage behavioural changes towards more conservation-friendly practices [22], BioBlitz events in particular have shown a variety of potential impacts on participants, including improved scientific knowledge and understanding of local wildlife, skill development (e.g. species identification), behavioural change as well as recreational fun [15, 16].

### The City Nature Challenge

The most prominent example of a large-scale BioBlitz event is the City Nature Challenge (CNC), an annual global initiative, focussing on urban biodiversity. Initially launched in 2016 as a friendly competition between Los Angeles and San Francisco, the CNC has since expanded significantly, with over 700 cities worldwide participating in 2024 [23]. The event encourages participants to document as many plants, animals, and fungi as possible within their urban surroundings and upload their observations to the online biodiversity data platform iNaturalist over consecutive days. Quality-checked observations are directly transferred to the Global Biodiversity Information Facility [24], the most comprehensive international data infrastructure providing open access to biodiversity data globally [25]. The CNC’s approach to data sharing is in line with best practice for BioBlitz events, ensuring that data is openly accessible, reusable and contributes to long-term biodiversity monitoring [15, 26]. Data generated through the CNC have already proven valuable for urban biodiversity research, e.g. CNC observations can reveal meaningful patterns of urban biodiversity and help examine biotic homogenization both within and between cities [27]. To evaluate the significance of BioBlitz events for the routine assessment of biodiversity, the capital of Germany, Berlin, appears to be a suitable study site. Since the 1970s, Berlin has been a global pioneer in the systematic research of urban habitats, land use types and biodiversity in cities [28, 29]. Berlin hosts an impressive diversity of urban flora and fauna, with 20,000–30,000 species of animals, fungi, and plants recorded within the city, e.g. including around 1,500 wild plant species, and 165 species of breeding birds [30, 31]. Additionally, Berlin provides habitat for various rare and endangered species of amphibians, reptiles, and insects [32]. However, until today Berlin lacks a comprehensive monitoring for species and habitat trends. Despite individual surveys for protected species [33], species groups (Red Lists), and habitats, there is no structured, comprehensive monitoring system in place [34].

As Citizen Science is known to have great potential in urban biodiversity research [7, 35] and the city of Berlin increasingly uses platforms like the “Artenfinder” [36] to gather biodiversity data, in this study we want to use the case of Berlin to determine if and how BioBlitz events like the CNC can support and complement existing biodiversity surveys and monitoring. We specifically focus on species composition, data quality, and participant engagement, three aspects which are also interconnected. In order to evaluate the contribution of the CNC Berlin to urban biodiversity monitoring in general, we analysed the species richness and composition in the CNC Berlin 2023 and 2024 datasets. As BioBlitz events are based on unstructured, opportunistic observations, biases towards certain taxa are likely [37–39].

Citizen Science projects rely on active public involvement, but the level and type of engagement, measured by the number of observations or time invested, can vary significantly [40, 41]. Following Nielsen [42], we hypothesize that few participants provide most of the total observations. We examine the influence of expert participation and targeted excursions on species composition and richness, and compare the CNC data to a spatially and temporally matched reference dataset in order to identify gaps and overrepresented groups. Another central aspect is data quality, assessed by whether observations achieve “Research Grade” status on iNaturalist. Understanding whether data quality is maintained under increased user engagement is essential to evaluate the potential for expanding future BioBlitz events. Additionally, we investigate whether observations of rare, endangered, specially protected or invasive species are included, as these records enhance the scientific and the dataset’s value for conservation issues.

## Materials and methods

### Data collection

The data used in this study were collected during two consecutive CNC events in Berlin, which took place from 28 April to 1 May 2023 and from 26 to 29 April 2024. Participants recorded their observations via the Citizen Science platform iNaturalist, uploading photographic or acoustic evidence of flora, fauna, and fungi alongside spatial and temporal metadata. We are aware that it is not possible to verify whether a user account on iNaturalist represents multiple people or a single person. In this study, we understand the term “participant” to refer to every user account that contributed observations to CNC Berlin 2023 or 2024.

### Preparing data

The raw data of the CNC Berlin 2023 and 2024 were exported from the Citizen Science platform iNaturalist on June 21, 2024. For the raw dataset, all observations were selected regardless of their quality grade or review status. For each individual observation, the information on the user account, the geodata, the taxon identification and higher taxonomic classifications were then exported. All of this information is available to download for free and without restrictions via iNaturalist. Observations of cultivated life forms, as well as observations without photographic or acoustic evidence or without geographic or temporal information, were excluded from further analysis. For subsequent analysis, we categorised observations into major taxonomic groups: “Amphibians”, “Arachnids”, “Birds”, “Chromista”, “Fungi and Lichens”, “Insects”, “Mammals”, “Molluscs”, “Plants”, “Reptiles”, “Viruses”, “Bacteria” and “Other Animals”.

### Data quality assessment

In this study, we assessed data quality based on the iNaturalist classification system, which distinguishes between different levels. The analyses in this paper were based on observations with the highest quality rating of “Research Grade” unless otherwise stated. An observation reaches “Research Grade” when it includes a photo or sound file, geographic coordinates, and a date, and when the proposed species identification is confirmed by at least one additional user.

### Participant contributions

We categorized participants into five activity levels based on the number of observations they submitted: “very low activity” (obs. = 1), “low activity” (1 < obs. ≤5), “medium activity” (5 < obs. ≤100), “high activity” (100 < obs. ≤1000) and “very high activity” (obs. >1000).

Our categorisation enabled us to analyse how effort (measured by the number of observations) relates to the diversity and quality of the submitted data. Additionally, by linking observations to user accounts, the contribution of individual experts can be identified. This information will be used to explain patterns in species composition across the dataset. It is important to emphasise that, due to the one-time data download, CNC years 2023 and 2024 had different time spans to reach “Research Grade”. This means that the years cannot be directly compared with each other, only the activity levels within each year. Particularly for the year 2024, it is therefore not yet possible to speak of reaching a plateau in research-grade observations.

### Species richness, species composition and taxonomic representation: CNC vs. GBIF reference dataset

To compare the species composition, species richness and taxonomic representation of CNC Berlin 2023 and 2024 with a reference dataset, we downloaded occurrence data for Berlin from the Global Biodiversity Information Facility (GBIF). The dataset was restricted to fully georeferenced, species-level identifications from within Berlin’s administrative boundaries and limited to the period from 15 April to 15 May for the years 2016 – 2022. After filtering, the GBIF reference dataset contained 81,270 observations [43]. In addition to this reference data set, the complete data set for Berlin between 1938 and 2022 was also downloaded under the same conditions. The GBIF reference dataset (*N* = 81,270 observations) includes observations from various Citizen Science observation platforms, including “iNaturalist.org” (*N* = 12,619, 16%), “Pl@ntNet” (*N* = 11,794, 15%), “Observation.org” (*N* = 992, 1%), “naturgucker.de” (*N* = 21,332, 26%), “ArtenFinder” (*N* = 4182, 5%), and the “Cornell Lab of Ornithology” (*N* = 30,352, 37%) (Figure A1, Appendix). While these platforms partly differ in scope and user base, all their data is collected by humans (experts and non-experts) and documented with photos or acoustic evidence and geographic data. Only occurrence records based on observational data were included; preserved specimens such as herbarium records were excluded to ensure comparability with CNC observations. The differences in the Taxonomic Representation between the CNC Berlin 2023 and 2024 datasets and the GBIF reference dataset in terms of the number of species per taxonomic group were analysed by calculating the relative frequency (RF in %) for CNC 2023, CNC 2024, and GBIF. Based on these values, we calculated and visualised the relative frequency difference (RFD in %) between the CNC Berlin 2023 and 2024 datasets and GBIF. This analysis shows the strengths and deviations of the 2023 and 2024 CNC events compared to the GBIF reference dataset, exhibiting the taxonomic focus of the observation dataset. The analysis also provides information on species groups that were less observed or uploaded during the CNC.

The relative frequency (RF) and difference values (RFD) were calculated using the following formulas:

$$R{F_{CNC}}\left( \% \right) = \frac{{Number\,of\,species\,in\,taxonomic - based\,group\,CNC}}{{Total\,number\,of\,species\,CNC}} \times 100$$

$$R{F_{GBIF}}\left( \% \right) = \frac{{Number\,of\,species\,in\,taxonomic - based\,group\,GBIF}}{{Total\,number\,of\,species\,GBIF}} \times 100$$

$$RFD\left( \% \right) = R{F_{CNC}} - R{F_{GBIF}}$$

This approach allows for a direct comparison of species group representation between datasets and was also used by Zhang et al. [44] to assess differences in iNaturalist datasets in terms of species composition.

Lastly, we checked the dataset for noteworthy findings of particularly protected or rare species, as well as those listed as invasive in Berlin.

### Software and tools

All analyses were conducted in R (version 4.0.1, R Core [45]), using the packages ggplot2 (version 4.0.1 [46]), and VennDiagram (version 1.7.3 [47]).

## Results

In 2023, a total of 12,218 observations were submitted for CNC Berlin. After data processing, 12,179 observations (99.7%) remained available for analysis. In 2024, 17,949 observations were uploaded, of which 17,852 (99.5%) were retained for analysis.

### Data quality

**Fig. 1:**
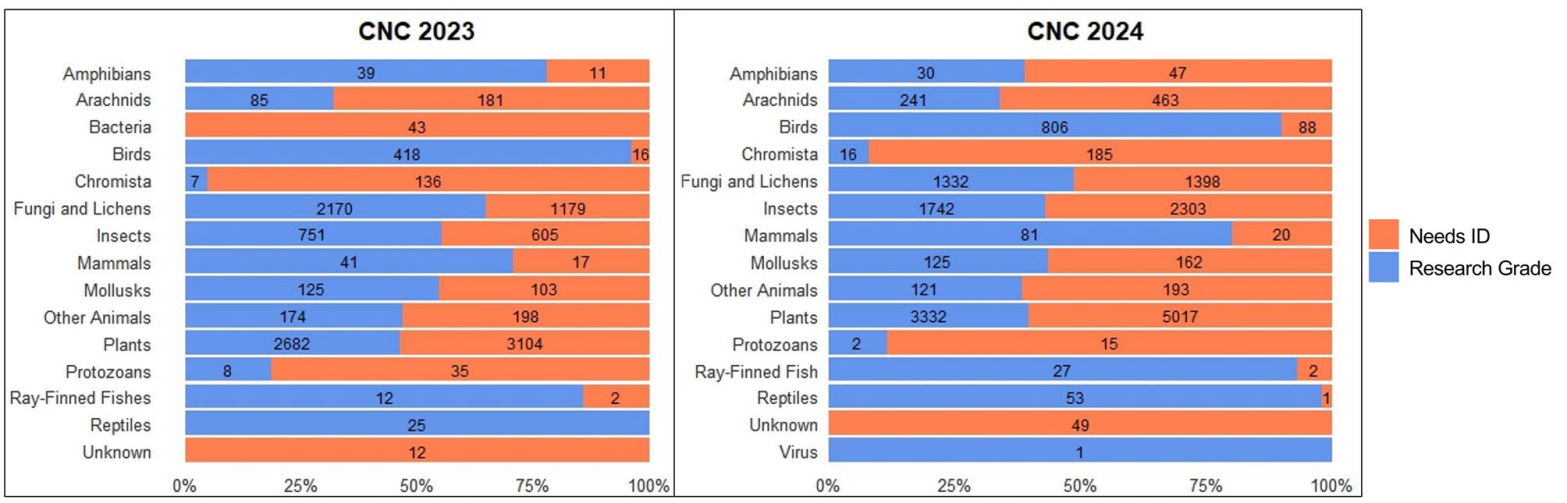
Percentage and number (in the bars) of observations per taxonomic group during the CNC Berlin 2023 (*N* = 12,179) and 2024 (*N* = 17,852). The colours code for observation quality categories. Note that the two years have to be viewed separately and cannot be compared to each other, due to the difference in evaluation time

At the time of data download, 54% of observations in 2023 and 44% in 2024 were classified as “Research Grade” (Table 1). Note, that both years have to be viewed separately and cannot be compared due to a different evaluation time. For both 2023 and 2024, the majority of “Research Grade” observations in numbers are “Plants”, “Fungi and Lichens”, and “Insects” (Fig. 1). In 2023, these groups accounted for 41%, 33%, and 11% of all “Research Grade” records, respectively. In 2024, “Plants” are dominant with 42% as well, followed by “Insects” (22%) and “Fungi and Lichens” (17%). In 2024, one virus achieved “Research Grade” status based on photographic evidence. For both years, vertebrate taxa like “Reptiles” and “Birds” show higher percentages of “Research Grade” then e.g. “Insects” and “Arachnids” (Fig. 1).

**Table 1:** Overview of the City Nature Challenge (CNC) 2023 and 2024 datasets with the raw data, as well as the amount and the quality of data that can be used after sorting the datasets. Data quality dynamically changes over time, so the given numbers refer to the date of downloading the data

dataset	timeframe	observations		data quality used observations	
		total	used	“Needs ID”	“Research Grade”
CNC 23	28.04. − 01.05.2023	12,218	**12,179**	5,642 (46%)	6,537 (54%)
CNC 24	26.04. − 29.04.2024	17,949	**17,852**	9,934 (56%)	7,909 (44%)

### Participant contribution

**Fig. 2:**
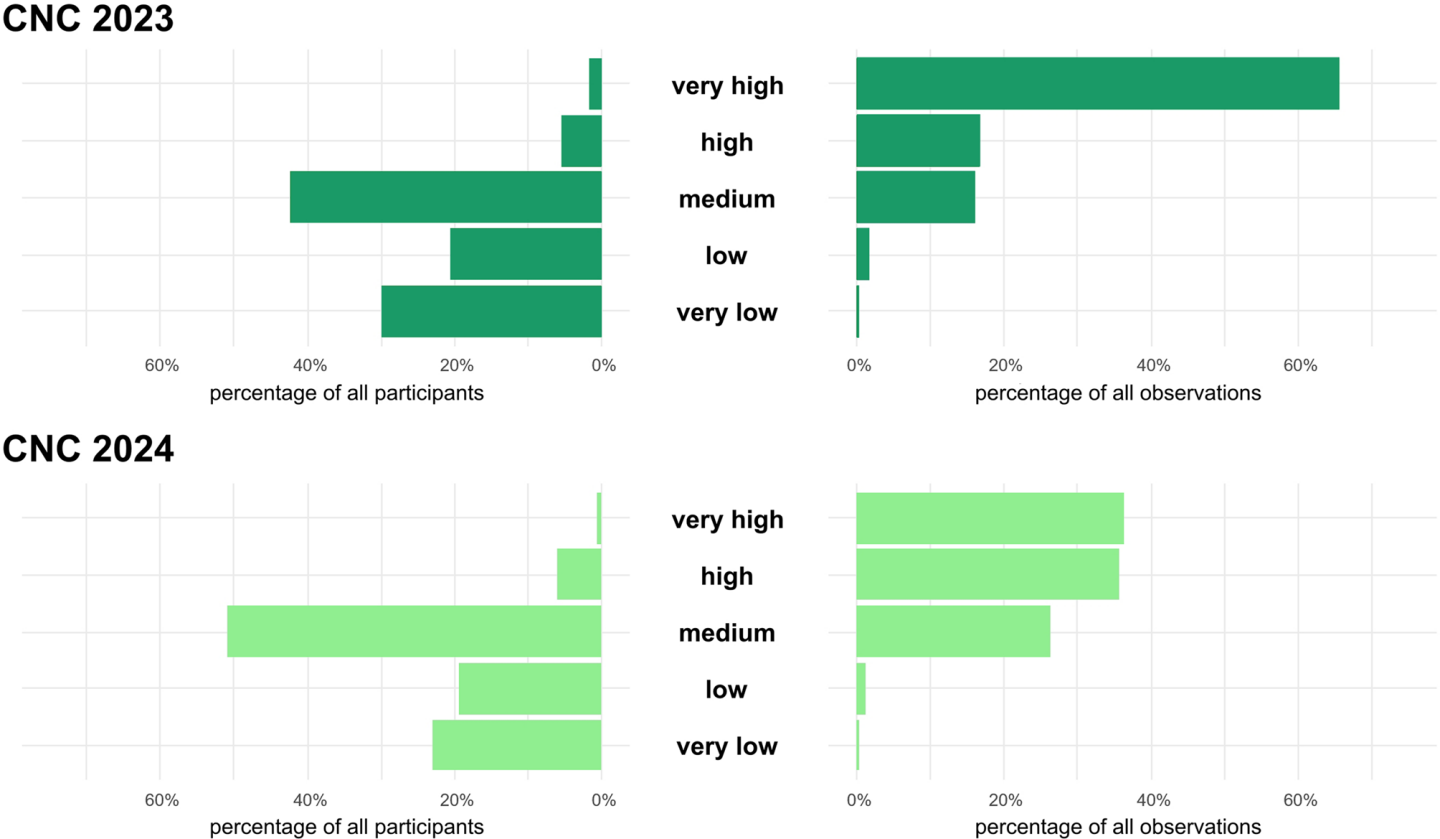
Contribution of the five different participant activity level groups to the dataset. Upper bars (dark green) represent the ratio of each group among all participants (*N* = 184) and all observations (*N* = 12,179) in 2023. Lower bars (bright green) represent the ratio among all participants (*N* = 361) and all observations (*N* = 17,853) in 2024

**Fig. 3:**
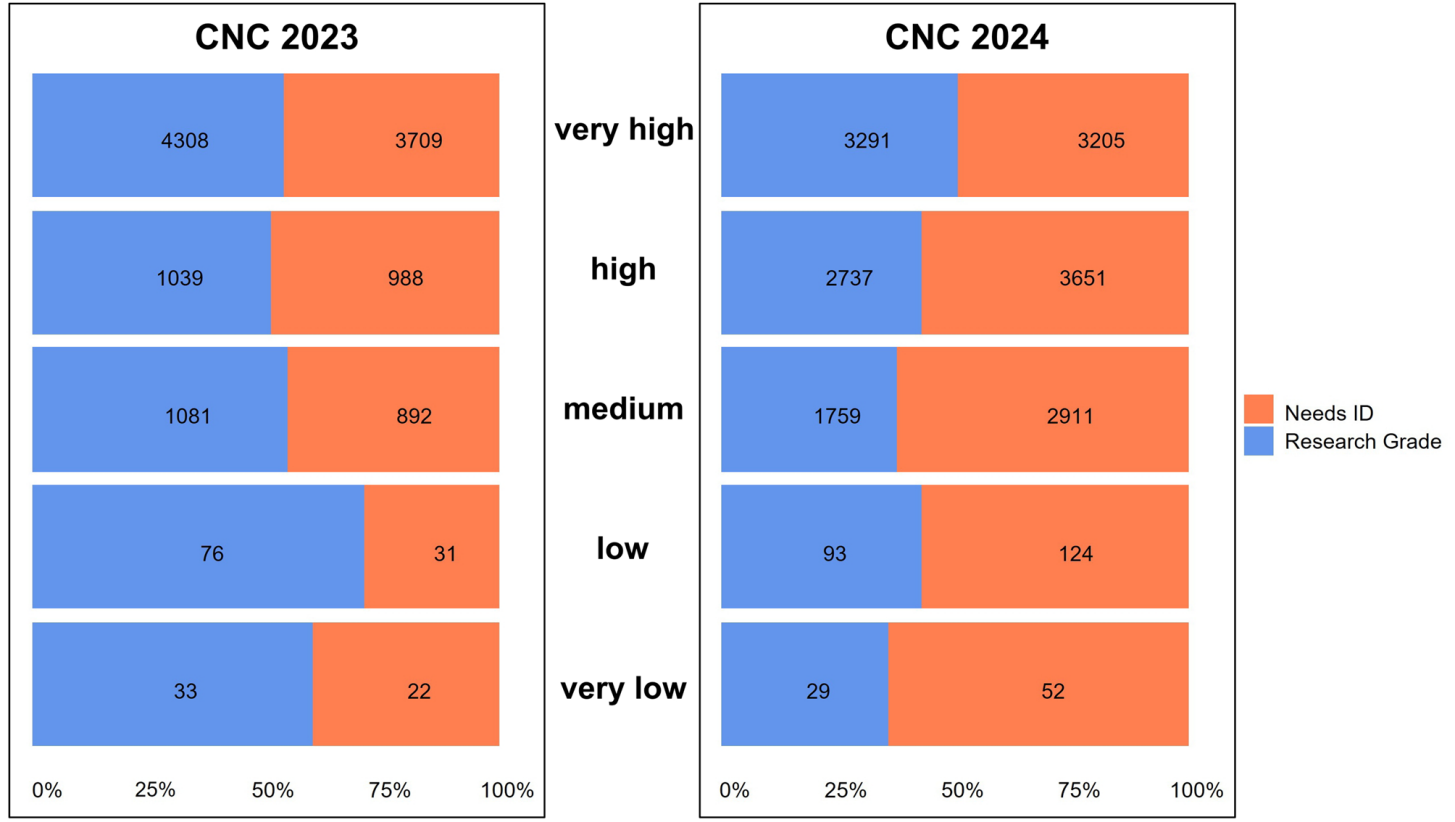
Proportion of observations classified asResearch Grade and Needs ID across five participant activity level groups during CNC Berlin 2023 (*N* = 12,179) and 2024 (*N* = 17,853). Note that the two years have to be viewed separately and cannot be compared to each other, due to the difference in evaluation time

The number of participants at CNC Berlin nearly doubled from 184 in 2023 to 361 in 2024. In both years, most participants showed “medium activity” (42% in 2023; 51% in 2024). The “very high activity” group remained marginal, with three participants in 2023 and two in 2024 (Fig. 2). Regarding data quality, the proportion of observations classified as “Research Grade” differed only moderately across activity levels. In 2023, percentages ranged from 52% to 71%, with the highest share observed in the “low activity” group. In 2024, the range shifted to 43% to 51%, and the “very high activity” group showed the highest proportion of “Research Grade” observations, being the only group to exceed the 50% threshold (Fig. 3).

**Fig. 4:**
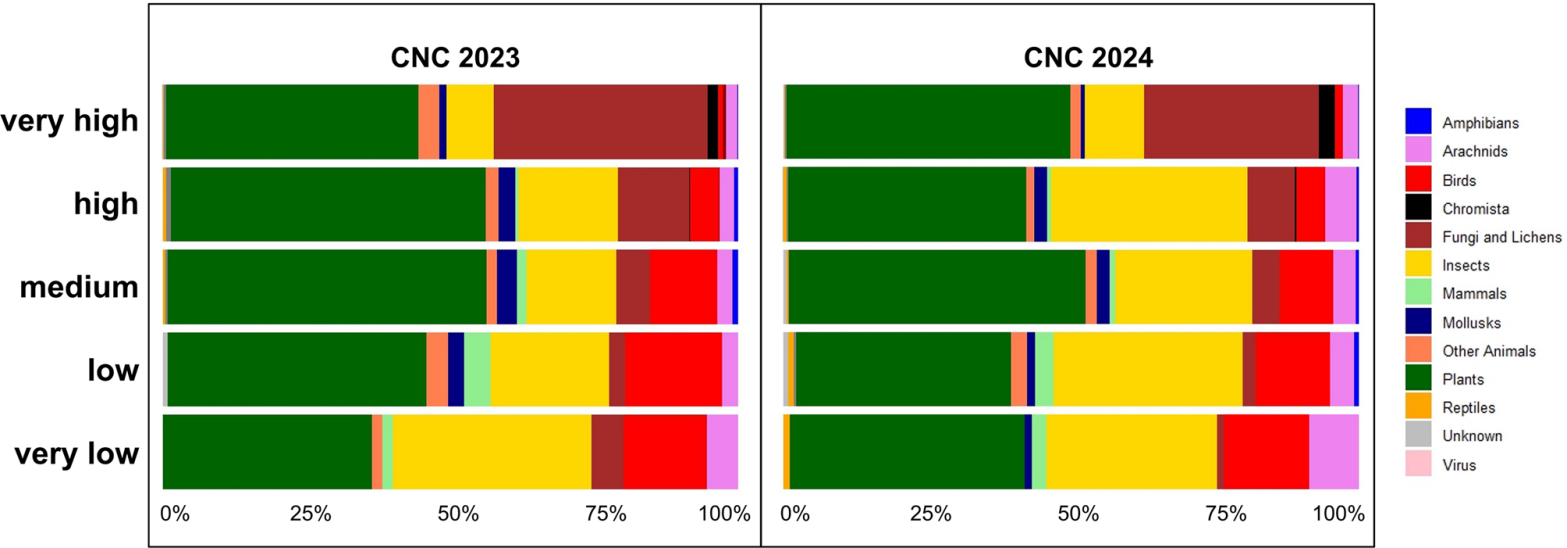
Taxonomic composition of observations across five participant activity level groups during CNC Berlin 2023 and 2024

Evaluating the observation groups according to taxonomic categories produced the following results: In both years of the challenge, most observations were made in the “Plants” and “Insects” categories. The exception in the distribution in both years were the groups of “Fungi and Lichens”, as well as “Birds”. Within the groups, the observations of birds decreased from “very low activity” to “very high activity”. In contrast, the observations of “Fungi and Lichens” increased from “very low activity” to “very high activity” (Fig. 4).

### Species richness, species composition and taxonomic representation: CNC vs. GBIF reference dataset

**Fig. 5:**
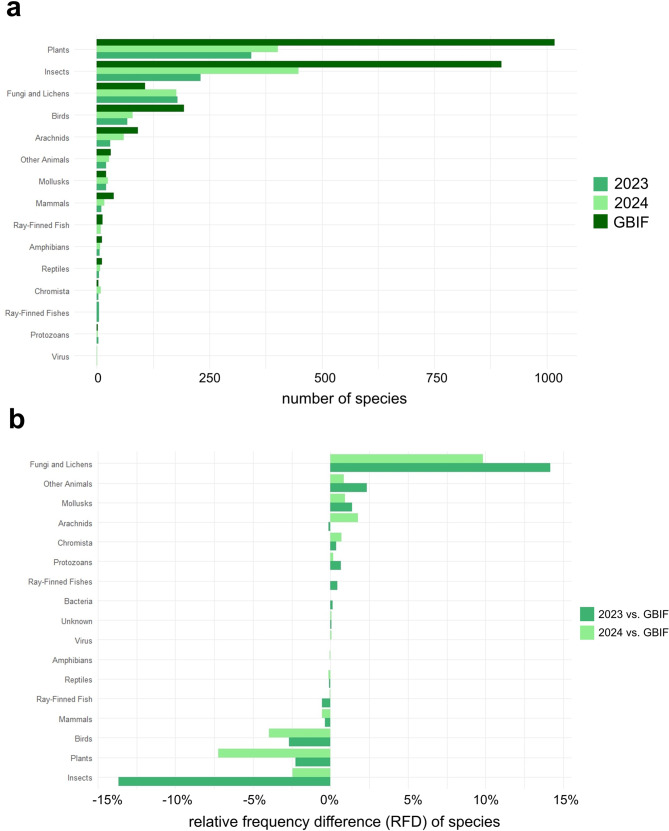
**a**) species richness across taxonomic groups in the CNC Berlin 2023 (*N* = 924), CNC Berlin 2024 (*N* = 1,267), and the GBIF reference dataset (*N* = 2,440). **b**) direct comparison of CNC datasets to the GBIF reference data by means of a relative frequency difference (RFD). Note the strong devation in fungi and lichens (positive) and insects (negative)

A total of 924 species were recorded in CNC Berlin 2023 and 1,267 species in 2024, with higher observed species richness in 2024. These findings were compared with our selections from the GBIF reference dataset, which includes 2,440 species. Plants (42%) and insects (37%) accounted for the largest share of species richness in the reference data, followed by birds (8%) (Fig. 5). In the CNC Berlin datasets “Plants” and “Insects” also dominated, followed by “Fungi and Lichens”. While plants (37%) showed the highest richness in 2023, insects (35%) accounted for the largest share in 2024.

**Fig. 6:**
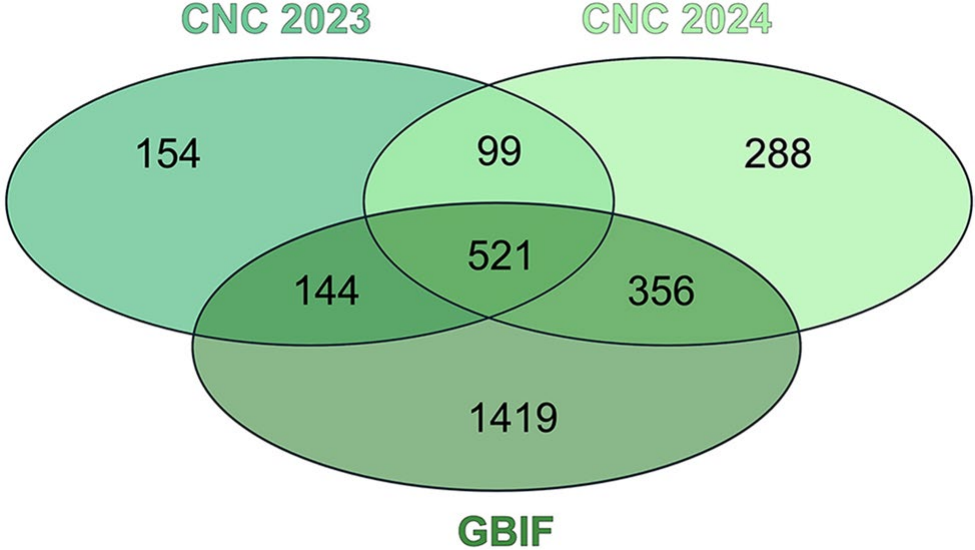
Venn diagram showing the overlap of species between the CNC Berlin 2023 (*N* = 924), CNC Berlin 2024 (*N* = 1,267) and the GBIF reference dataset Berlin (*N* = 2,440)

Considering the relative frequency difference (RFD) in terms of species numbers between the two years of the CNC compared to the GBIF dataset for Berlin, the largest positive deviation is in the fungi and lichens category in 2023 (10%) and 2024 (15%). The largest negative deviation for 2023 is calculated for insects (−14%) and 2024 for plant species (−7%) (Fig. 5). The Venn diagram (Fig. 6) shows an overlap in the species found during the two years CNC in comparison to the GBIF reference dataset of 1,027 (42%) species, while 1,413 species were only recorded in the GBIF dataset. Looking at the species that only occur in the GBIF reference dataset, the three highest numbers of species are 600 plant species, 561 insect species and 108 bird species that were not reported during the two CNC events. A total of 541 (18%) species were found during the CNC Berlin 2023 and 2024 but have not been included before in the GBIF reference dataset for Berlin. The GBIF total dataset for Berlin contained 899,288 observations with 6,755 species. In relation to the complete GBIF dataset for Berlin (1938–2022), CNC Berlin recorded 264 species in 2023 and 2024 that had not previously been listed.

**Fig. 7:**
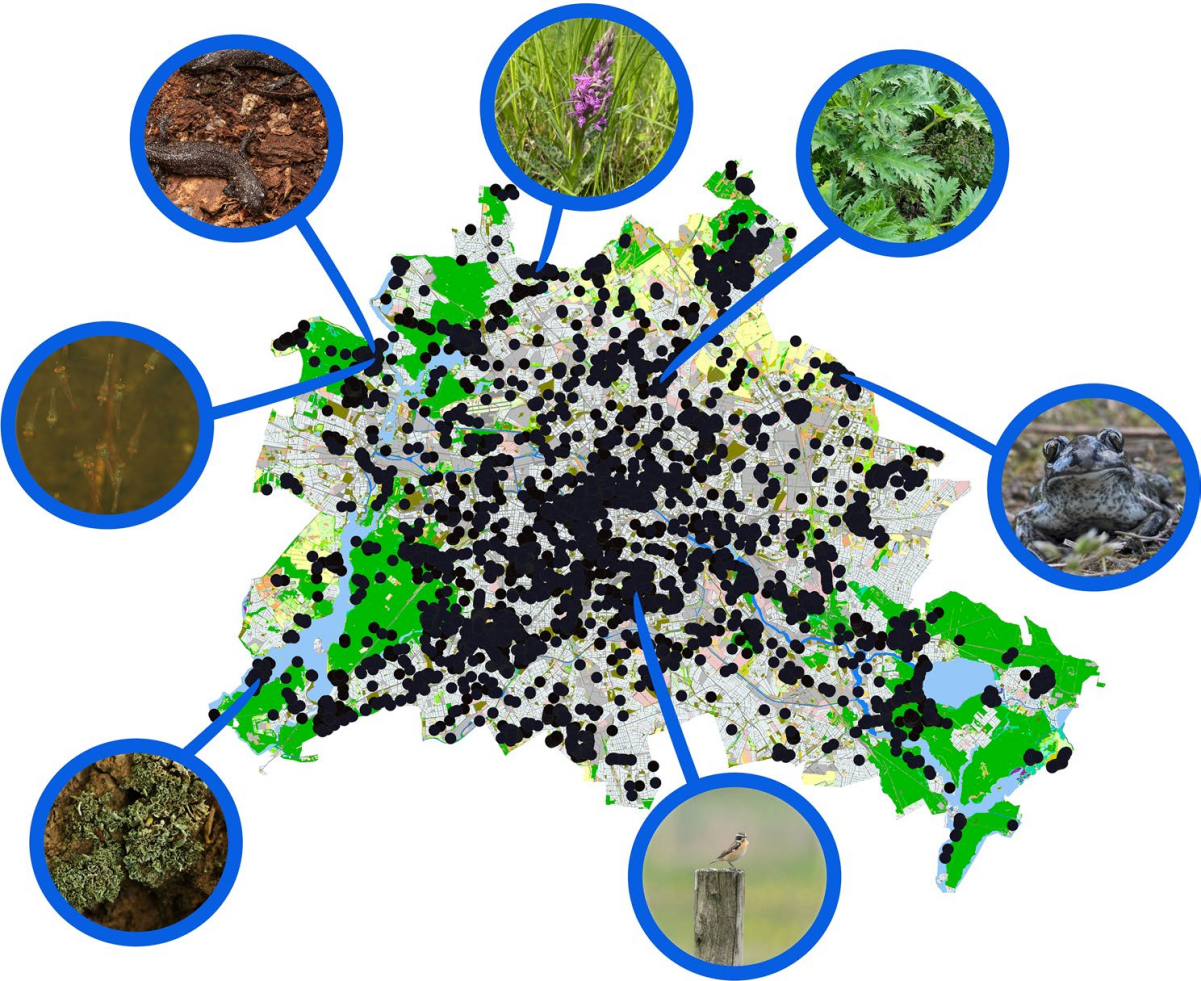
Map of Berlin including all observations of the City Nature challenges in 2023 and 2024 (black dots). Highlighted are seven examples of rare or protected species, documented during these two BioBlitz events. Clockwise from top left: Triturus cristatus (photo: frederic_griesbaum, with permission), Dactylorhiza majalis (photo: astridtorr, CC-BY-NC), Heracleum mantegazzianum (photo: fjalbrecht, CC-BY-NC), Pelobates fuscus (photo: alexis_orion, with permission), Saxicola rubetra (photo: mathildebessertnettelbeck, with permission), Cladonia digitata (photo: frederic_griesbaum, with permission), Hemimysis anomala (photo: alexis_orion, with permission)

During CNC Berlin 2023 and 2024, observations included threatened species of amphibians like the Spadefoot Toad (*Pelobates fuscus*) and the Great Crested Newt (*Triturus cristatus*), rare birds like the Whinchat (*Saxicola rubetra*), and several invasive species such as Crayfish (*Procambarus spp*.) and Giant Hogweed (*Heracleum mantegazzianum*). A potentially invasive mysid (*Hemimysis anomala*) was recorded 2024 for the first time in Berlin (Fig. 7).

## Discussion

This study is the first analysis of City Nature Challenge data from Germany, and one of the first systematic analyses of BioBlitz data in general. While there are publications of CNC data from Austria [48, 49] and the US [27], other recent studies focused more on CNC organizers’ [50] and participants’ [41] behaviour.

First of all, numbers of participants, observations and the species were higher in 2024 than in 2023 (Table 1, Fig. 2). Compared to Berlin’s previous participation in the CNC in 2018, 2019 and 2022, there was a particular increase in the number of observations, rising from 122 in 2018 to 3,003 in 2019 and reaching 4,788 in 2022. The number of species also increased from 84 species in 2018 to 596 in 2019 and 886 species in 2022. The number of participants increased from 20 in 2018 to a spectacular 732 in 2019, falling slightly to 123 in 2022 [51]. Also considering the participant activity levels measured by the uploaded observations, clear differences were identified. There was a lower number of participants with very high activity for both years, but they contributed a large proportion of the total observations. This variation in the observation or user activity levels was also found by other studies on the iNaturalist overall dataset [52] and other Citizen Science projects [40, 53]. For the CNC Berlin dataset, the proportion of observations from “very high activity” observers among the total dataset was successfully reduced from over 60% in 2023 to less than 40% in 2024, which also means that more people contributed more than a few observations (Fig. 2). Regarding data quality, we would like to emphasise once again that it is difficult to compare the number of “Research Grade” observations in our data between the two years, as 2023 had more time for evaluation than 2024. As of 11 December 2025, proportions of “Research Grade” observations have again changed for 2023 (increase from 54% to 56%) as well as for 2024 (increase from 44% to 51%) [54, 55].

Nevertheless, it is notable that within years, data quality differs only slightly between activity levels. In 2023, the percentage of “Research Grade” observations is even the highest in the “low activity” group (Fig. 3). On one hand, this could be explained because beginners which only upload a few observations might also show a bias towards common and easy to identify species. On the other hand, this result aligns with earlier findings, demonstrating that even untrained or novice observers can produce reliable biodiversity data using platforms such as iNaturalist [56]. It underscores that high-quality contributions - such as observations reaching Research Grade status - are achievable regardless of participants’ prior experience and highlights the scientific value and inclusiveness of broad public engagement in BioBlitz events.

The comparison of the uploaded data from the CNC Berlin 2023 and 2024, divided into taxonomic groups, reveals the influence of very enthusiastic species specialists, who contribute a high number of observations and thus strongly influence the overall dataset. The number of observations in the category “Fungi and Lichens” increases with the observer activity level in both years of the CNC Berlin. Especially in the category “very high activity”, i.e., a group with only a few participants, the proportion differs particularly compared to the other participant groups. In 2023, for example, the *Botanic Garden and Botanical Museum Berlin* explicitly offered a tour on lichens in the centre of Berlin [57]. The mycological society “*Pilzkundliche Arbeitsgemeinschaft Berlin-Brandenburg e.V.”* also participated in CNC 23 and 24 and contributed to a large number of fungi observations. In relation to the total CNC observation dataset, Fungi and Lichen observations accounted for 27% in 2023 and 20% in 2024, making them the second-largest taxa group in 2023 and the third-largest in 2024. A similar pattern was reported in Palma et al. [58], in which the participation of the Entomological Society of Victoria in several events certainly contributed to the large contribution of insect records during the CNC in Melbourne in 2021. On the one hand, this discloses a possible bias in species composition of BioBlitz datasets, created by relative overfocussing on certain taxa. On the other hand, this example shows how motivating species experts to join in BioBlitz events can lead to provision of data of particularly underrepresented taxa groups by Citizen Science. To understand why, where and when people take part in the CNC BioBlitz, a short pre-post survey could be distributed at the start of the tours or sent online to participants to find out how they learned about the challenge in future years and how they contributed data, especially for the CNC Berlin 2025 [59].

The results of the data quality analysis, categorized by taxonomic groups, reveal clear differences. Groups such as birds, reptiles, fish, and mammals are more likely to achieve “Research Grade” status compared to arachnids, protozoans, insects, and chromists (Fig. 1). This could be due to a low species knowledge of latter groups, but also because of technical limitations of identification from photos. It is known from research that larger organisms with clear morphological characteristics are identified more reliably on iNaturalist [60, 61] than small species that are difficult to identify taxonomically, e.g., dissection or a microscope is often required to identify lichens [62]. During the City Nature Challenges Berlin less than half of the insect observations achieved “Research Grade” status. For BioBlitz events in general and the future CNC Berlin, it may be beneficial to involve species experts from both an academic and non-academic background with a high level of species knowledge [16] to join data collection and species identification. In addition, differences in “Research Grade” proportions between years are likely influenced by unequal validation time rather than reflecting inherent differences in data quality.

Of the 2,440 species listed on GBIF from 15 April 2016 to 15 May 2022, 1,027 species were also found during the two CNC Berlin events in 2023 and 2024, representing 42% of the species listed on GBIF. Even within the short observation period of the CNC, a considerable proportion of the species recorded in GBIF for Berlin during the reference period could be documented. Notably, 541 species were recorded during CNC Berlin 2023 and 2024 that were not previously included in the GBIF reference dataset. When comparing the proportion of species with the GBIF reference dataset, it becomes clear that more fungi, lichens, and mollusc species were uploaded during the CNC Berlin 2023 and 2024, while the GBIF reference dataset contains a higher proportion of insect, plant, and bird species (Fig. 6). Even when compared with the complete GBIF dataset for Berlin, CNC Berlin was able to add an impressive 264 species in 2023 and 2024 that had not previously been listed on GBIF. With regard to species composition, plant and insect species account for the highest proportions of the total number of species in both the GBIF and CNC Berlin 2023 and 2024 datasets. This finding is not surprising, as both categories have a high species diversity in Berlin (see [30] for plants), and plants are generally easier to document photographically due to their static nature at specific locations. Nevertheless, the species’ numbers of the relative frequency difference values of the CNC Berlin plants differ from those on GBIF (Fig. 6). This suggests that the CNC data sets may contain fewer plant species than the GBIF data set. For future CNC Berlin projects, this is a valuable indication that plants should also be included in the observations in order to reflect the existing urban biodiversity.

Our analysis showed that in both years, the CNC documented many protected and endangered species in Berlin. Non-native and invasive species were also regularly observed in both years. These observations suggest that, in addition to records of widespread species, findings of high conservation relevance were also made. This aligns with previous research highlighting the potential of Citizen Science to contribute meaningfully to nature conservation and biodiversity research [8, 63] and biases in the composition of taxonomic species during the CNC [27]. Moreover, data from various BioBlitz events and formats have already been associated with conservation efforts, particularly in relation to the detection and monitoring of non-native, invasive, or potentially invasive species [15, 19, 64, 65]. BioBlitz events may serve as early warning tools, offering initial evidence of the presence of such species and prompting more systematic monitoring efforts. A particularly notable and scientifically relevant example is the first recorded observation of the non-native Bloody-red Mysid (*Hemimysis anomala*) in Berlin, documented via the iNaturalist platform (https://www.inaturalist.org/observations/212866330). This species, native to the Ponto-Caspian region, is currently the subject of intensive research at Lake Stechlin in Brandenburg [66]. Its detection in Berlin now provides a basis for conservation authorities to initiate further investigations and, if necessary, implement appropriate management measures. These findings highlight the potential of BioBlitz events to contribute to the detection of rare, endangered, or newly occurring species.

Several limitations should be considered. First, unequal validation time possibly affected comparisons between years. Second, the GBIF dataset represents heterogeneous citizen science data rather than standardized monitoring. Third, BioBlitz data are inherently shaped by observer behaviour and sampling bias. Data from unstructured, opportunistic BioBlitz events such as the worldwide CNC, differs from classic systematic monitoring methods. The observation data collected in the CNC Berlin and other opportunistic data are often characterised by various biases, such as the subjective object selection of the observers, the choice of observation site, uneven sampling methods and the choice of observation time and period [37–39]. As the CNC is based on unstructured, non-replicable observations and does not represent a systematic survey of existing biodiversity, the documented species diversity depends on the interests and expertise of the participants. Species experts contribute in particular to the groups for which they have specific expertise, which can influence the species composition. Eye-catching or particularly interesting species (e.g., flowering plants, birds) are more often photographed by participants, which can also shift the recorded species diversity [67]. Characteristics of animals, plants, and fungi also influence how well they can be documented. Animal species with a long flight distance, such as some birds or mammals, are more difficult to document, even if they occur frequently. The same applies to small and highly mobile species such as ants, which are often underrepresented on species reporting platforms such as iNaturalist because they are difficult to record [48]. Studies related to the iNaturalist dataset emphasise the need to analyse and adjust biases in Citizen Science data on a case-by-case basis. For a solid assessment of biodiversity, more detailed modelling of the observation process is needed to better understand the impact of user behaviour on biodiversity estimates. Such modelling is crucial for developing tools that can be used to harness unstructured Citizen Science observations for biodiversity research questions [52, 68].

For science and nature conservation, data from BioBlitz events and Citizen Science in general have been shown to support standardised monitoring programs and can potentially fill research gaps [7]. While we still recommend standardised scientific monitoring of urban environments, we herein emphasize the opportunity to detect non-native or invasive species, as well as new or unknown localities for rare and protected species by means of BioBlitz events. Eventually, we encourage other researchers to investigate large BioBlitz datasets such as those generated by the City Nature Challenge in other parts of the world or even comprehensively on a larger geographic scale.

## Electronic supplementary material

Below is the link to the electronic supplementary material.

Supplementary Material 1

Supplementary Material 2

Supplementary Material 3

## Data Availability

The datasets supporting the findings of this study are available in the GBIF repository (https://doi.org/10.15468/DL.C4WRPP; https://doi.org/10.15468/dl.aqs55z) and on iNaturalist (https://www.inaturalist.org).

## Abbreviations

CNC: City Nature Challenge
CODATA: Committee on Data of the International Science Council
CS: Citizen Science
GBIF: Global Biodiversity Information Facility
RF: Relative Frequency
RFD: Relative Frequency Difference
