# Supplementary material for: The City Nature Challenge as an urban BioBlitz: evaluating Citizen Science contributions to biodiversity monitoring in Berlin
Source: BMC Ecol Evol. 2026 May 20;26:49. doi: 10.1186/s12862-026-02524-w (PMC13188247; doi:10.1186/s12862-026-02524-w)
Supplement: Supplementary file 1 — Supplementary Material 1 [file 12862_2026_2524_MOESM1_ESM.docx]

*
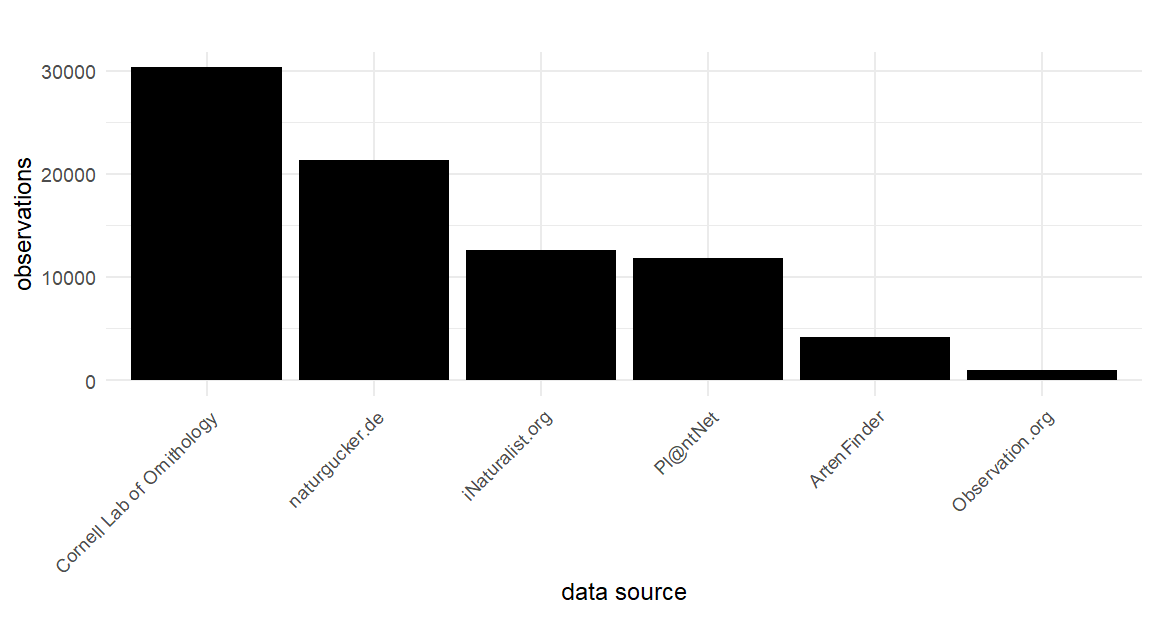
*

*Figure A1: Data sources of the GBIF reference dataset for Berlin (N= 81,270), showing the total number of observations per source.*
