# Supplementary material for: The City Nature Challenge as an urban BioBlitz: evaluating Citizen Science contributions to biodiversity monitoring in Berlin
Source: BMC Ecol Evol. 2026 May 20;26:49. doi: 10.1186/s12862-026-02524-w (PMC13188247; doi:10.1186/s12862-026-02524-w)
Supplement: Supplementary file 2 — Supplementary Material 2 [file 12862_2026_2524_MOESM2_ESM.docx]

*Table A1: Total number of observations from the City Nature Challenge (CNC) Berlin 2023, divided into taxonomic-based groups. The table shows the numbers of all recorded observations, the number of identified species per taxonomic-based group, and the number of participating observers and identifiers (as of June 21, 2024).*
